# Msh2 Blocks an Alternative Mechanism for Non-Homologous Tail Removal during Single-Strand Annealing in *Saccharomyces cerevisiae*

**DOI:** 10.1371/journal.pone.0007488

**Published:** 2009-10-16

**Authors:** Glenn M. Manthey, Nilan Naik, Adam M. Bailis

**Affiliations:** 1 Division of Molecular Biology, Beckman Research Institute, City of Hope National Medical Center, Duarte, California, United States of America; 2 Scripps College Post-Baccalaureate Premedical Program, Claremont, California, United States of America; National Cancer Institute, United States of America

## Abstract

Chromosomal translocations are frequently observed in cells exposed to agents that cause DNA double-strand breaks (DSBs), such as ionizing radiation and chemotherapeutic drugs, and are often associated with tumors in mammals. Recently, translocation formation in the budding yeast, *Saccharomyces cerevisiae*, has been found to occur at high frequencies following the creation of multiple DSBs adjacent to repetitive sequences on non-homologous chromosomes. The genetic control of translocation formation and the chromosome complements of the clones that contain translocations suggest that translocation formation occurs by single-strand annealing (SSA). Among the factors important for translocation formation by SSA is the central mismatch repair (MMR) and homologous recombination (HR) factor, Msh2. Here we describe the effects of several *msh2* missense mutations on translocation formation that suggest that Msh2 has separable functions in stabilizing annealed single strands, and removing non-homologous sequences from their ends. Additionally, interactions between the *msh2* alleles and a null allele of *RAD1*, which encodes a subunit of a nuclease critical for the removal of non-homologous tails suggest that Msh2 blocks an alternative mechanism for removing these sequences. These results suggest that Msh2 plays multiple roles in the formation of chromosomal translocations following acute levels of DNA damage.

## Introduction

Exposure to ionizing radiation and other DNA damaging agents is associated with the appearance of chromosomal aberrations in a variety of systems [1], [2], [3], [4]. Among these are chromosomal translocations that appear frequently enough to be useful in estimating initial exposure levels [5]. Recently, in the budding yeast, *Saccharomyces cerevisiae*, a dose of ionizing radiation sufficient to cause hundreds of DSBs per genome were found to result in high frequencies of formation of non-reciprocal translocations by HR between dispersed repetitive sequences [6]. Given the ubiquity of repetitive sequences in the human genome [7], this mechanism may also contribute to the translocations observed in the lymphocytes of people following acute exposure to ionizing radiation [5], [8], [9].

Creation of DSBs elicits two general repair responses in eukaryotic systems [10], [11], [12], [13], [14]; HR, which requires interaction between sequences at or near the break and homologous sequences lying on a sister-chromatid, homolog or non-homologous chromosome, and non-homologous end-joining (NHEJ), which utilizes minimal or no homology to bring together the ends of broken DNA molecules. While both mechanisms play important roles in the repair of DSBs in these systems, HR is the dominant determinant of radiation resistance in budding yeast [15], [16], [17], whereas NHEJ plays a greater role in mammalian cells [18], [19]. Chromosomal DSBs generated by the action of the yeast mega-endonucleases HO [20] and I-SceI [21] have been used to simulate the effects of exposure to ionizing radiation in yeast [22], [23] and mammalian [10], [24] cells. Consistent with studies of cells exposed to ionizing radiation [4], [6], [25], [26], [27], [28], endonuclease-generated DSBs have been shown to result in the formation of a variety of genome rearrangements by both HR and NHEJ. These rearrangements include deletions [23], [29], [30], insertions [31], [32], and translocations [28], [33], [34], [35], [36], [37], [38], [39].

Simulating the circumstances following acute ionizing radiation exposure in yeast, we have recently shown that multiple enzyme catalyzed DSBs adjacent to repetitive 60 or 300 bp sequences promotes the generation of chromosomal translocations in 1 to 10% of wild-type diploid cells [38], [39]. This high frequency of translocation formation correlates closely with the frequency observed in yeast exposed to sufficient radiation to generate an average of 250 randomly distributed DSBs per genome, with at least seven of these statistically likely to occur in repetitive sequences [6]. Patterns of substrate utilization and genetic control observed for the endonuclease catalyzed events suggests that these rearrangements are the result of SSA [38], [39], a non-conservative mechanism of HR [12], [40], [41].

Among the factors critical for translocation formation by SSA is Msh2 [38], the central MRR factor [42], [43] that has also been implicated in multiple HR mechanisms [44], [45], [46]. Msh2, and its companion protein, Msh3 [47], [48], bind to a variety of branched DNA structures [49], [50], including the intersection between double-stranded DNA (dsDNA) and single-stranded DNA (ssDNA). Such structures are created upon annealing complementary sequences on 3′ ssDNA molecules produced by the exonucleolytic processing of DSBs adjacent to repetitive genomic sequences [45], [51]. Binding of the Msh2-Msh3 heterodimer at these junctions is thought to stabilize the dsDNA intermediates [46], [52] such that a suite of proteins, including the structure-specific nuclease, Rad1-Rad10 [45], [53], [54], [55], [56], the endonuclease subunit and checkpoint signaling protein, Slx4 [57], [58], [59], and the Rad1-Rad10 recruitment factor, Saw1 [60], can act to remove the non-homologous 3′ ssDNA tails in advance of covalent joint formation [51].

Investigating the function of Msh2 in both MMR and non-homologous tail removal, Studamire et al. [61] identified a series of *msh2* missense alleles that confer dominant-negative effects on MMR and a range of effects on the retention of a plasmid by intra-molecular gene conversion following linearization by HO-endonuclease [45]. In the current study, the effects of four of these alleles on translocation formation by SSA were determined. One allele conferred a similar defect in translocation formation regardless of whether the 60 or 300 bp recombination substrates were used, while two others conferred defects with the shorter but not the longer substrates. This suggests that Msh2 has length-dependent and -independent functions in translocation formation by SSA. Surprisingly, combining the *msh2* alleles with a null allele of *rad1* resulted in variable suppression of the profound translocation defect conferred by *rad1*Δ**. These data are consistent with Msh2 playing multiple roles in translocation formation by SSA; stabilizing dsDNA intermediates, facilitating the removal of non-homologous tails from dsDNA intermediates by Rad1-Rad10, and blocking an alternative mechanism for tail removal. This study supports the conclusion that Msh2 makes a major contribution to the genome instability that arises following the generation of many chromosome breaks.

## Results

### Loss of *MSH2* confers a major defect in translocation formation initiated by simultaneous DSBs adjacent to recombination substrates on different chromosomes

**Figure 1:**
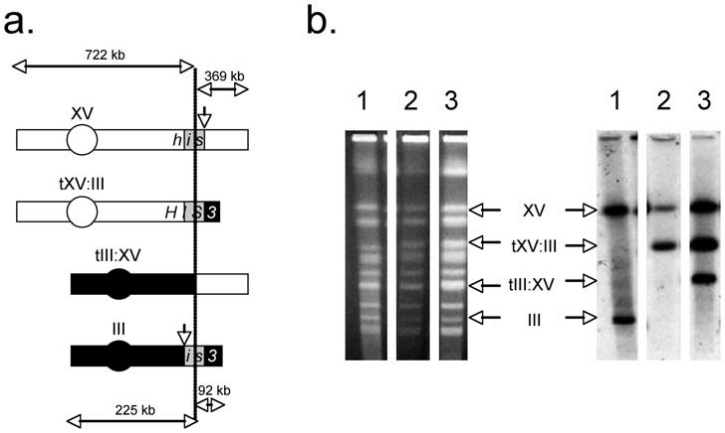
Detection of translocation chromosomes in His^+^ recombinant diploids. (a) Expected chromosomal products of HR between *his3* substrates. The *his3-*Δ*3′* substrate is located at the *HIS3* locus on one copy of chromosome XV. A *his3-Δ200* allele on the other copy of chromosome XV (not pictured) lacks sufficient homology to generate an intact *HIS3* coding sequence by recombination with either *his3-*Δ*3′* or *his3-*Δ*5′*. A *his3-*Δ*5′* substrate that shares either 60 bp or 300 bp of sequence with *his3-*Δ*3′* (gray boxes) is located at the *LEU2* locus on one copy of chromosome III. Following DSB formation at the HO cut sites, indicated by downward facing arrows that flank the right side of the homology box in *his3-*Δ*3′* and the left side of the homology box in *his3-*Δ*5′*, the homologous sequences can interact to create an intact *HIS3* coding sequence on a tXV:III translocation chromosome. The tIII:XV reciprocal translocation chromosome, which is created by a process that utilizes little homology between the broken ends [38] may also appear. (b) CHEF gels and blots of chromosomes prepared from His^−^ parent and His^+^ recombinant strains. Pictured on the left are chromosomes that had been separated by CHEF on agarose gels and were stained with ethidium bromide and photographed. Pictured on the right are gel-separated chromosomes that have been denatured in alkali, blotted to nylon, hybridized with a ^32^P-labeled 1.8 kb *Bam*HI genomic clone containing the *HIS3* coding sequence, and autoradiographed. Lanes: (1) His^−^ parent. (2) His^+^ recombinant carrying the tXV:III translocation chromosome. (3) His^+^ recombinant carrying the tXV:III translocation chromosome and the tIII:XV reciprocal translocation chromosome.

We previously demonstrated that simultaneous HO endonuclease-catalyzed DSBs adjacent to appropriately oriented 60 or 300 bp repetitive sequences on different chromosomes in wild-type diploid cells results in the appearance of chromosomal translocations in 1 to 10% of the survivors (Figure 1, Table 1) [38], [39]. This process, referred to as T2, utilizes two truncated copies of the *HIS3* gene that share 60 or 300 bp homologous segments [38]. A 5′ truncated allele (*his3-*Δ*5′*) is incorporated into the *LEU2* locus on chromosome III and substitutes a 117 bp fragment of the *MAT* locus carrying the recognition sequence for HO endonuclease [20] for the 5′ end of the *HIS3* gene. A 3′ truncated allele (*his3-*Δ*3′*) lies at the *HIS3* locus on chromosome XV and substitutes the HO cut-site sequence for the 3′ end of the gene. The *HIS3* locus on the other copy of chromosome XV carries the *his3-*Δ*200* allele [62], which lacks sufficient homology to produce an intact *HIS3* coding sequence through interaction with either *his3* recombination substrate. The *his3* substrates have the same orientation with respect to their centromeres such that HR between them will not form dicentric chromosomes. HO endonuclease is provided from a galactose-inducible HO gene inserted into the *TRP1* locus on chromosome IV. Importantly, deletion of one [38], or both (G. Manthey and A. Bailis, unpublished observations) copies of the *MAT* locus was found not to significantly affect the efficiency of T2. This suggests that neither the expression of the *MAT* genes, nor their repair after HO endonuclease-mediated DSB formation (Table 2) affect the efficiency of T2.

**Table 1:** Effect of loss of *MSH2* on frequencies of spontaneous DRR and translocation formation by HR following a DSB adjacent to one or both recombination substrates^a^.

Genotype^b^	DRR^c^	T1^d^		T2^e^	
		60 bp	300 bp	60 bp	300 bp
Wild type	4.6×10^−4^	1.1×10^−6^	1.3×10^−5^	9.3×10^−3^	6.5×10^−2^
	[1]	[1]	[1]	[1]	[1]
	(3.8, 6.1)	(0.9, 1.3)	(0.9, 1.8)	(8.4, 14.0)	(5.5, 9.0)
*msh2*Δ	1.9×10^−4^	1.6×10^−6^	2.2×10^−5^	2.3×10^−4^	5.3×10^−4^
	[0.4]	[1.5]	[1.7]	[0.025]	[0.008]
	(1.3, 2.7)	(1.4, 1.8)	(1.8, 2.8)	(1.3, 2.6)	(5.5, 9.0)

a Median frequencies were determined from a minimum of 10 independent cultures for each strain. Fold differences from the median frequency observed with wild type strains are indicated in brackets. The 95% confidence intervals are indicated in parentheses.

b Frequencies of DRR were determined with wild type and *msh2::hisG* mutant haploid strains. Frequencies of translocation formation were determined with homozygous wild type and *msh2::hisG-URA3-hisG/msh2::hisG-URA3-hisG* mutant diploid strains.

c Frequencies of spontaneous DRR between 415 bp direct repeats at the *HIS3* locus in haploid cells were determined as described in the Materials and Methods, and previously (75).

d Frequencies of translocation formation in diploid cells by HR between a *his3-*Δ*5′* substrate at the *LEU2* locus on one copy of chromosome V and a *his3-*Δ*3′* substrate at the *HIS3* locus on one copy of chromosome XV were determined following a HO endonuclease-mediated break adjacent to the *his3-*Δ*5′* substrate as described in the Materials and Methods, and previously (38). *his3-*Δ*5′* substrates that shared either 60 bp or 300 bp of homology with the *his3-*Δ*3′* substrate were used. The frequencies observed in wild type strains were reported previously (38).

e Frequencies of translocation formation in diploid cells by HR between *his3-*Δ*5′* and *his3-*Δ*3′* following HO endonuclease-mediated break adjacent to both substrates were determined as described in the Materials and Methods, and previously (38). *his3-*Δ*5′* substrates that shared either 60 bp or 300 bp of homology with the *his3-*Δ*3′* substrate were used. The frequencies observed in both wild type *msh2*Δ*/msh2*Δ mutant strains were reported previously (38).

**Table 2:** Plating efficiencies of wild-type and mutant diploids before and after the formation of multiple HO-catalyzed DSBs^a^.

	Plating efficiency (%)	
Genotype	Pre-induction	Post-induction
Wild-type	92	69
	(64, 103)	(32, 94)
*msh2*Δ*/msh2*Δ	48	37
	(35, 70)	(17, 51)
*msh3*Δ*/msh3*Δ	79	59
	(69, 93)	(48, 67)
*msh6*Δ*/msh6*Δ	83	65
	(73, 89)	(48, 68)
*rad1*Δ*/rad1*Δ	87	58
	(61, 109)	(28, 70)
*msh2*Δ*/msh2*Δ* rad1*Δ*/rad1*Δ	46	24
	(36, 87)	(18, 29)

a Median plating efficiencies before and after expression of HO endonuclease were determined as described in the Materials and Methods. The 95% confidence intervals are indicated in parentheses.

Characterization of His^+^ T2 recombinants by genomic Southern and chromosome blotting showed previously that all contained the novel 0.78 Mb chromosome consistent with the presence of the tXV:III translocation formed by HR between the *his3-*Δ*3′* and the *his3-*Δ*5′* substrates (Figure 1)[38], [39]. From 13 to 27% of these were also found to carry the 0.59 MB tIII:XV reciprocal translocation chromosome that is generated by rejoining the HO cut chromosome fragments by precisely ligating the HO cut ends (G. Manthey and A. Bailis, unpublished observations). Since the presence of the tIII:XV translocation is not selectable in diploids the frequency of appearance in recombinants may not be indicative of its frequency of generation.

**Figure 2:**
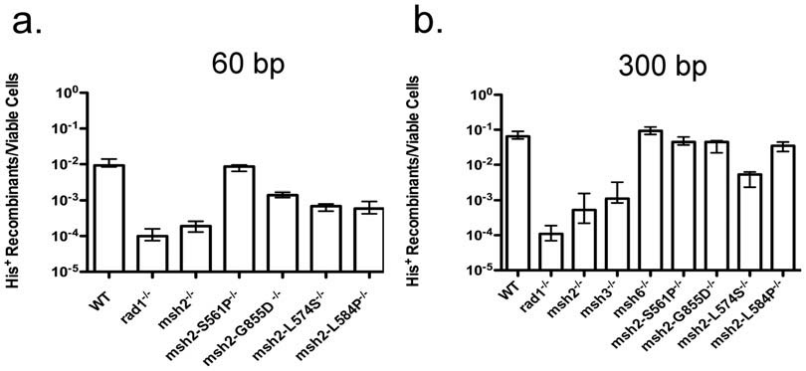
Frequencies of translocation following DSB formation by HO endonuclease adjacent to both *his3* substrates in homozygous wild-type and mutant diploids. (a) Frequencies of His^+^ colony formation by HR between 60 bp substrates. Frequencies of His^+^ colony formation from a minimum of 10 trials with each strain were determined as discussed in the Materials and Methods. Median frequencies are presented. Error bars represent 95% confidence intervals determined as described in the Materials and Methods. (b) Frequencies of His^+^ colony formation by HR between 300 bp substrates.

It was also shown previously that Msh2 is a critical factor in T2 as the frequency of translocation formation is reduced 40- to 125-fold in *msh2*Δ*/msh2*Δ-null homozygotes (Table 1, Table S1, and Figure 2) [38]. This, along with the requirement for *RAD1*, *RAD52* and *RAD59*, and the dispensability of *RAD51* are consistent with these translocations occurring by SSA [12], [38], [45]. SSA has also been proposed to contribute to the formation of deletions by both spontaneous [40], [41] and HO-stimulated [23] recombination between non-tandem, direct repeats (direct-repeat recombination – DRR), which also require *MSH2* (Table 1) [44], [45]. Similar to DRR, T2 has been shown to require *MSH3* but not *MSH6*, as translocation frequency in the *msh3*Δ*/msh3*Δ homozygote is reduced 58-fold, which is not significantly different from the 120-fold reduction in the *msh2*Δ*/msh2*Δ** homozygote, while the frequency in the *msh6*Δ*/msh6*Δ homozygote is not significantly different from wild-type (Figure 2B). This is consistent with translocation formation by SSA utilizing the Msh2-Msh3 but not the Msh2-Msh6 heterodimer. Interestingly, translocation formation stimulated by a DSB adjacent to only one substrate (T1) has a significant but minimal requirement for *MSH2* (Table 1), consistent with previous evidence that T1 and T2 proceed by distinct mechanisms [38]. The loss of *MSH2* and *MSH3* also minimally affected the repair by gene conversion of HO-mediated DSBs at the *MAT* loci on both copies of chromosome III as plating efficiency following induction of the expression of HO was not significantly affected (Table 2).

### The phenotypes conferred by *msh2* missense alleles suggest involvement of Msh2 in homologous sequence length-dependent and –independent aspects of translocation formation by SSA

We further explored the role of *MSH2* in T2 by determining the effects of a set of four *msh2* missense alleles that had been previously characterized with respect to their impact on MMR, as well as the removal of non-homologous tails from the ends of a linear plasmid during reclosure by intra-molecular gene conversion [61]. While these alleles conferred a range of defects in non-homologous tail removal, from no defect to a defect equivalent to that conferred by the *msh2*-null allele, all had severe, dominant-negative effects on mismatch repair. This indicates that the roles of *MSH2* in MMR and non-homologous tail removal are genetically separable. All of the mutations map to the region of *MSH2* that encodes the highly conserved C-terminus of the Msh2 protein that contains several functionally important domains [47], [63], [64], [65]. Two of them alter amino acids that are conserved from yeast to humans, consistent with their considerable impact on MMR.

The variable ability to remove non-homologous tails during gene conversion conferred by the missense alleles suggested that they might have similar effects on the efficiency of T2, which also requires the removal of non-homologous tails. Consistent with the results obtained by Studamire et al. [61] the allele that conferred the least effect on intra-molecular gene conversion also had the least effect on T2. The *msh2-S561P* allele, which alters a serine that is not conserved phylogenetically was found to have no significant effect on translocation frequencies regardless of substrate length (Figure 2, Table S1). This result suggests that, like the function of Msh2 in intra-molecular gene conversion, its function in T2 is genetically separable from its function in MMR. Also, similar to previous results, the *msh2-L574S* allele, which mutates a conserved leucine and confers an intermediate defect in intra-molecular gene conversion resulted in an intermediate 12- to 13-fold reduction in T2 frequency that was significantly different from the frequencies observed in wild type and *msh2*-null mutant cells regardless of substrate length. Importantly, the level of expression of *msh2-L574S*, and the stability of its protein product were previously shown to be similar to those of the wild-type *MSH2* allele [61], suggesting that this general reduction in translocation frequency was not due to a decrease in the level of the protein. Distinct from the previous alleles, the *msh2-L584P* allele, which alters a leucine that is not conserved phylogenetically and which confers a defect in intra-molecular gene conversion that is as severe as that conferred by a *msh2-*null allele, resulted in a substantial, 16-fold reduction with the 60 bp substrates, but a minimal, 2-fold reduction in the frequency of T2 with the 300 bp substrates, both of which were significantly different from those observed in wild type and *msh2*Δ-null mutant cells. Similarly, the *msh2-G855D* allele, which alters a conserved glycine residue implicated in the ATPase activity of the Msh2-Msh6 heterodimer [65], and which confers a slight defect in intra-molecular gene conversion [61], resulted in a substantial, 7-fold reduction in translocation with the 60 bp substrates but a minimal, 1.4-fold reduction in translocation with the 300 bp substrates, both of which were significantly different from those observed in wild type and *msh2*Δ-null mutant cells. These results suggest that the roles of *MSH2* in intra-molecular gene conversion and T2 are distinct. Further, it indicates that *MSH2* is involved in sequence length-dependent and –independent aspects of translocation formation by SSA.

**Figure 3:**
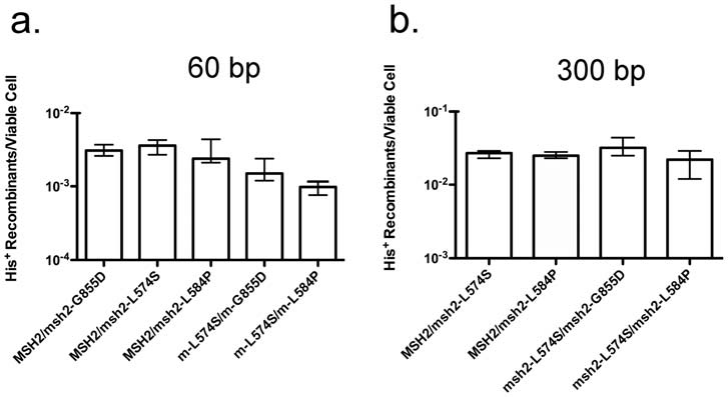
Frequencies of translocation following DSB formation by HO endonuclease adjacent to both *his3* substrates in heterozygous diploids. (a) Frequencies of His^+^ colony formation by HR between 60 bp substrates. Median frequencies and confidence intervals were determined as described in the legend to Figure 2 and in the Materials and Methods. (b) Frequencies of His^+^ colony formation by HR between 300 bp substrates.

The defects conferred by the *msh2-L574S*, *msh2-L584P* and *msh2-G855D* alleles were explored further by evaluating their dominance/recessiveness relationships with the wild-type *MSH2* allele. The frequencies of T2 with the 60 bp substrates in the *MSH2/msh2-L574S*, *MSH2/msh2-L584P*, and *MSH2/msh2-G855D* heterozygotes were 2.6-, 3.9- and 3.0-fold lower than wild-type respectively (Figure 3A). While the frequencies in the heterozygotes are all significantly different from those of their respective single mutant homozygotes and wild-type (Table S1) they suggest that all three mutant alleles are largely recessive to the wild-type allele. The *msh2-L574S* allele was also essentially recessive to wild-type for use of the 300 bp substrates, as the *MSH2/msh2-L574S* heterozygote displayed a frequency of T2 that, while significantly different from wild-type was only 2.4-fold lower (Figure 3B, Table S1). These dominance/recessiveness relationships suggest that the intermediate defects in translocation formation conferred by the *msh2-574S*, *msh2-584P* and *msh2-G855D* alleles are due to losses of function in Msh2.

In addition to their relationships with the wild-type allele, dominance/recessiveness relationships among *msh2-L574S*, *msh2-L584P* and *msh2-G855D* were also determined. Both *msh2-L584P* and *msh2-G855D* were dominant to *msh2-574S* with respect to T2 with the 300 bp substrates as the *msh2-L574S/msh2-L584P* and *msh2-L574S/msh2-G855D* heterozygotes displayed 2-and 3-fold reduced frequencies (Figure 3B) that were not significantly different from the 2- and 1.4-fold reduced frequencies in the *msh2-L584P/msh2-L584P* and *msh2-G855D/msh2-G855D* homozygotes but were significantly different from the 12-fold decreased frequency observed in the *msh2-L574S/msh2-L574S* homozygote (Figure 2B, Table S1). Similarly, *msh2-G855D* is dominant to *msh2-L574S* with respect to T2 with the 60 bp substrates as the 6-fold reduced frequency of translocation in the *msh2-L574S/msh2-G855D* heterozygote (Figure 3A) was not significantly different from the frequency in the *msh2-G855D/msh2-G855D* homozygote and significantly different from the 13-fold reduced frequency in the *msh2-L574S/msh2-L574S* homozygote (Table S1). Combining the *msh2-L574S* and *msh2-L584P* alleles resulted in a 12-fold reduced frequency of T2 with the 60 bp substrates that was not significantly different from the 13- and 16-fold reduced frequencies observed in the *msh2-L574S/msh2-L574S* and *msh2-L584P/msh2-L584P* homozygotes (Figure 2A, Table S1), suggesting neither dominance nor recessiveness. Although the *msh2-L584P* and *msh2-G855D* alleles confer defects in translocation formation that are dependent on the length of the substrates, while the defect conferred by the *msh2-L574S* allele is not (Figure 2), their patterns of complementation are most consistent with these mutations leading to defects in the same sequence of events.

### Epistasis interactions between *msh2* missense alleles and a null allele of *rad1* suggests variable ability to engage an alternative mechanism for removal of non-homologous tails during translocation formation by SSA

**Figure 4:**
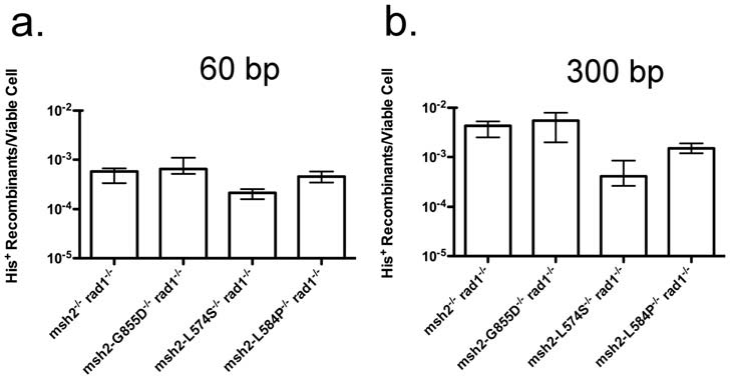
Frequencies of translocation following DSB formation by HO endonuclease adjacent to both *his3* substrates in double homozygotes. (a) Frequencies of His^+^ colony formation by HR between 60 bp substrates. Median frequencies and confidence intervals were determined as described in the legend to Figure 2 and in the Materials and Methods. (b) Frequencies of His^+^ colony formation by HR between 300 bp substrates.

In our previous study of the genetic control of T2 we observed that the *rad1*Δ*-* and *rad59*Δ**-null alleles individually reduced translocation frequency with the 60 bp substrates by 60- to 90-fold, but that the translocation frequency in the *rad1*Δ*/rad1*Δ* rad59*Δ*/rad59*Δ double homozygotes was reduced only 3-fold [38]. This is consistent with the notion that Rad1 and Rad59 work together to execute non-homologous tail removal during translocation formation by SSA [52], but that an alternative pathway for removal of non-homologous tails is obscured when one is present and the other is absent [38]. Since Msh2-Msh3 has also been implicated in the facilitation of non-homologous tail removal by Rad1-Rad10 nuclease [50], [51], [52], [61], [66], [67], we examined the interaction between a null allele of *rad1* and the null and missense alleles of *msh2* with respect to T2. Previously, null alleles of *msh2* and *rad1* were found to be epistatic to one another with respect to deletion formation by spontaneous DRR [44] and the repair of a DSB by intra-molecular gene conversion [45]. In contrast, the *msh2*Δ and *rad1*Δalleles exhibited substantial mutual suppression with respect to T2 in *msh2*Δ*/msh2*Δ* rad1*Δ*/rad1*Δ** double homozygotes. Translocation frequencies were only 15- and 16-fold reduced from wild-type with both the 60 bp and 300 bp substrates (Figure 4), which were significantly different from the 40- and 120-fold reduced levels in the *msh2*Δ*/msh2*Δ** homozygotes and 90- and 600-fold reduced levels in the *rad1*Δ*/rad1*Δ** homozygotes (Table 1, Table S1, Figure 2). These results recall the relationship between the *rad1*Δ and *rad59*Δ alleles described previously [38], and suggest that Msh2 and Rad1 obscure an alternative pathway for non-homologous tail removal during translocation formation by SSA.

Since the *msh2-L574S*, *msh2-L584P* and *msh2-G855D* alleles conferred defects in translocation formation that were distinct from those of the *msh2*Δ allele, we examined their effects when combined with the *rad1*Δ allele. Of the three alleles we found that *msh2-L574S* was the least capable of suppressing the effect of *rad1*Δ as the *msh2-L574S/msh2-L574S rad1*Δ*/rad1*Δ double homozygotes displayed a 72-fold reduced frequency of translocation with the 60 bp substrates (Figure 4A) that was not significantly different from the 90-fold reduced frequency observed in the *rad1*Δ*/rad1*Δ homozygote (Table 1, Table S1, and Figure 2), and a 160-fold reduced frequency with the 300 bp substrates (Figure 4B) that was close to but significantly different from the 600-fold reduced frequency displayed by the *rad1*Δ*/rad1*Δ homozygote. The *msh2-L584P* allele displayed a greater ability to suppress *rad1*Δ as the *msh2-L584P/msh2-L584P rad1*Δ*/rad1*Δ double homozygote displayed a 21-fold reduced frequency with the 60 bp substrates that was not significantly different from the frequencies in the *msh2-L584P/msh2-L584P* homozygote and *msh2*Δ*/msh2*Δ* rad1*Δ*/rad1*Δ double homozygote, and a 43-fold reduced frequency with the 300 bp substrates that was significantly different from both. The *msh2-G855D* allele suppressed *rad1*Δ to the greatest extent as translocation frequencies were 14-fold reduced with the 60 bp substrates and 15-fold reduced with the 300 bp substrates in the *msh2-G855D/msh2-G855D rad1*Δ*/rad1*Δ double homozygotes, which were not significantly different from those displayed by the *msh2*Δ*/msh2*Δ* rad1*Δ*/rad1*Δ** double homozygotes. These results further differentiate the *msh2* missense alleles with respect to translocation formation, and suggest that Msh2-Msh3 limits an alternative mechanism for non-homologous tail removal during SSA in the absence of Rad1-Rad10.

## Discussion

Following our initial observation that *MSH2* plays a critical role in generating chromosomal translocations by SSA [38], we investigated its function in greater detail. Like its role in deletion formation by SSA [52], [61], [67], Msh2 was found to work in collaboration with Msh3 but not Msh6 in generating translocations by T2 (Figure 2). This is consistent with previous work suggesting that the Msh2-Msh3 heterodimer is important in the removal of non-homologous tails during HR [46], [50]. The implication of Msh2-Msh3 in T2 led us to examine the effects of four *msh2* missense alleles that had been previously shown to confer a range of defects in non-homologous tail removal during HR [61]. These alleles had effects that suggest that Msh2 affects homologous sequence length-dependent and –independent aspects of translocation formation. The *msh2* missense alleles also displayed different abilities to suppress the profound defect in T2 conferred by *rad1*Δ**, suggesting that they result in differential engagement of a Rad1-Rad10-independent mechanism for non-homologous tail removal. Together, these observations indicate that Msh2-Msh3 controls multiple aspects of translocation formation by SSA.

Three of the missense alleles, *msh2-L574S*, *msh2-L584P* and *msh2-G855D*, conferred levels of translocation formation by T2 that were intermediate to the levels observed in wild-type and *msh2*Δ*/msh2*Δ** homozygotes, consistent with partial losses of function. Interestingly, while the *msh2-L574S* and *msh2-L584P* alleles led to similar 13- and 16-fold reductions in the frequency of T2 with the 60 bp substrates (Figure 2A), they had very different effects with the 300 bp substrates, resulting in 12- and 2-fold reductions, respectively (Figure 2B). This suggests that *msh2-L574S* imposes a defect in translocation formation by SSA that is relatively independent of the length of the substrates while *msh2-L584P* exerts its effect on SSA primarily when the substrates are short. We speculate that this may be related to the observation by Alani and colleagues that the Msh2-Msh3 heterodimer both binds to and alters the conformation of the junction between 3′ ssDNA and dsDNA, and that this conformational change may facilitate cleavage by Rad1-Rad10 [50]. The phenotype conferred by the *msh2-L574S* allele (Figure 2) may be explained if it leads to Msh2-Msh3 heterodimers that can execute the substrate length-dependent step of junction stabilization at wild-type levels, but perform the substrate length-independent conformational change at reduced levels. In contrast, *msh2-L584P* may exert its substrate length-dependent effect because it results in Msh2-Msh3 heterodimers whose binding cannot sufficiently stabilize the junctions formed by annealing short 60 bp ssDNA sequences, but can stabilize the junctions formed by annealing longer 300 bp sequences. Alternatively, these mutant Msh2-Msh3 heterodimers may impose a defect in annealing short ssDNA sequences [46]. Once the joints are stabilized, however, these mutant Msh2-Msh3 heterodimers can execute the conformational change that facilitates Rad1-Rad10 cleavage at normal levels. The phenotype conferred by the *msh2-G855D* allele is similar to, but less severe than that conferred by *msh2-L584P* (Figure 2), consistent with Msh2-Msh3 being somewhat less defective for annealing or junction stabilization. These results suggest that the function of Msh2-Msh3 in junction binding and stabilization is genetically separable from its function in facilitating cleavage by Rad1-Rad10.

The dominance/recessiveness relationships between *msh2-L574S*, and *msh2-L584P* and *msh2-G855D* support these notions (Figure 3). The dominance of *msh2-L584P* over *msh2-L574S* with respect to translocation with the 300 bp substrates suggests that Msh2-L584P-Msh3 heterodimers may replace Msh2-L574S-Msh3 heterodimers if they fail to make the conformational change. This would result in stable 3′ ssDNA/dsDNA junctions and efficient cleavage by Rad1-Rad10. Observing the same frequency of translocation formation with the 60 bp substrates in the *msh2-L574S/msh2-L584P* heterozygote as in the *msh2-L574S/msh2-L574S* and *msh2-L584P/msh2-L584P* homozygotes is consistent with neither form of Msh2-Msh3 heterodimer being able to function fully at the junctions between 3′ ssDNA and dsDNA. Replacement of one form of Msh2-Msh3 by the other at junctions in the heterozygote would leave the cell defective for translocation formation by SSA. The dominance of *msh2-G855D* over *msh2-L574S* with respect to translocation formation with both substrates (Figures 2 and 3) is also consistent with replacement of Msh2-Msh3 heterodimers that cannot execute the conformational change by those that can. However, in the heterozygotes containing the 60 bp substrates, this replacement results in the 3′ssDNA/dsDNA junctions possessing the same intermediate stability as in the *msh2-G855D/msh2-G855D* homozygote. It is important to note that although the *msh2-L574S* allele appears to permit the expression of steady-state levels of Msh2 that are comparable to wild-type [61], the phenotypes of the *msh2-L574S/msh2-L584P* and *msh2-L574S/msh2-G855D* heterozygotes may also be explained if Msh2 encoded by *msh2-L584P* and *msh2-G855D* form more stable associations with Msh3 than that encoded by *msh2-L574S*, thereby contributing to a higher percentage of Msh2-Msh3 heterodimers.

Rad1-Rad10 has long been recognized as an important component of SSA [29], [38], [44], [68], [69], [70] as it is required for the removal of non-homologous 3′ tails. Msh2-Msh3 is thought to play an important, but variable role in this process by binding, stabilizing, and optimizing the 3′ ssDNA/dsDNA junction for cleavage by Rad1-Rad10 [38], [45], [46], [50], [51], [52], [61], [66]. Previously, genetic interactions between *msh2-* and *rad1-*null alleles have been consistent with Msh2-Msh3 and Rad1-Rad10 working together in a single pathway for the removal of non-homologous tails during SSA [44], [45]. Therefore, it was with considerable surprise that we observed that the frequencies of T2 in *msh2*Δ*/msh2*Δ* rad1*Δ*/rad1*Δ double homozygotes were three- to 120-fold higher (Figure 3) than in the *msh2*Δ*/msh2*Δ and *rad1*Δ*/rad1*Δ homozygotes (Figure 2), consistent with Rad1-Rad10 and Msh2-Msh3 blocking a second, less efficient mechanism for removal of non-homologous tails. As discussed above, we had previously observed a similar interaction between *rad1*Δ*-* and *rad59*Δ*-*null alleles [38], consistent with the *RAD59* gene product working together with Rad1-Rad10 in establishing the major pathway for tail removal [52], and in blocking an auxiliary pathway. Recent experiments have shown that the *msh2*Δ*-* and *rad59*Δ-null alleles are epistatic to one another with respect to T2 (N. Pannunzio and A. Bailis, unpublished observations), suggesting that Rad59 may work together with Msh2-Msh3 in supporting Rad1-Rad10 in the main pathway of non-homologous tail removal, and, perhaps in blocking the auxiliary pathway. It is tempting to speculate that the auxiliary pathway may be related to a Rad1-Rad10- and Msh2-Msh3-independent pathway identified by Paques and Haber [71] for the removal of non-homologous tails shorter than 30 bp during HO endonuclease-stimulated intra-molecular gene conversion. If so, it may be substantially more capable of addressing non-homologous tails of more considerable length during translocation formation by SSA than it is during DSB-stimulated gene conversion or deletion formation.

The effects of the *msh2-L574S*, *msh2-L584P*, *msh2-G855D* and *msh2Δ* alleles on T2 suggested that they might display distinct epistasis relationships with the *rad1*Δ allele. In contrast to the *msh2*Δ allele, *msh2-L574S* displayed little capacity to suppress *rad1*Δ, as the frequencies of translocation in the *msh2-L574S/msh2-L574S rad1*Δ*/rad1*Δ** double homozygous strains were the same as, or less than 4-fold higher (Figure 4) than those in the *rad1*Δ*/rad1*Δ** single homozygotes (Figure 2), indicating that *rad1*Δ** is essentially epistatic to *msh2-L574S*. As this is comparable to the effect of expressing the wild-type *MSH2* allele, this suggests that *msh2-L574S* permits Msh2-Msh3 to block access to the machinery that executes alternative tail removal by binding in a wild-type fashion to the 3′ ssDNA/dsDNA junction. This is consistent with the previous data suggesting that *msh2-L574S* may affect the ability of Msh2-Msh3 to execute the conformational change that elicits cleavage of 3′ssDNA/dsDNA junctions by Rad1-Rad10 but may not affect junction binding by Msh2-Msh3. In contrast, the *msh2-L584P* and *msh2-G855D* alleles displayed significantly greater ability to suppress the effects of the *rad1*Δ allele as *msh2-L584P/msh2-L584P rad1*Δ*/rad1*Δ double homozygotes had five- to 12-fold higher frequencies of translocation (Figure 4) than *rad1*Δ*/rad1*Δ single homozygotes (Figure 2), while the *msh2-G855D/msh2-G855D rad1*Δ*/rad1*Δ double homozygotes had six- to 40-fold higher frequencies (Figure 4). These results are similar to those observed in *msh2*Δ*/msh2*Δ* rad1*Δ*/rad1*Δ double homozygotes where there is no Msh2, suggesting that *msh2-L584P* and *msh2-G855D* weaken the ability of Msh2-Msh3 to block the non-homologous tails from the action of the auxiliary pathway. This supports the data described earlier that suggest that *msh2-L584P* and *msh2-G855D* reduce the ability of Msh2-Msh3 to bind to 3′ ssDNA/dsDNA junctions. Alternatively, these epistasis results may reflect changes in an as yet unidentified function of Msh2-Msh3, perhaps one that is performed in conjunction with Rad59.

The interaction between *MSH2*, *RAD1* and *RAD59* described above is consistent with these genes encoding factors that function together to remove 3′ non-homologous tails during T2. We envision a scenario where Msh2-Msh3 and Rad59 act together to stabilize the 3′ ssDNA/dsDNA junction. Msh2-Msh3 then undergoes a conformational change that facilitates the binding of Rad1-Rad10 that cleaves the non-homologous tails, promoting extension of the 3′ ends by DNA polymerase, followed by ligation to create a covalent joint. In the absence of this ensemble, an as yet unidentified auxiliary apparatus processes the non-homologous tails, facilitating reduced levels of translocation formation by SSA. Future work will focus on further exploring these events at the molecular level.

## Materials and Methods

### Yeast strain and plasmid construction

Yeast strains (Table S2) were grown and genetically manipulated using established techniques [72]. All strains used in this study were isogenic with W303-1A [73]. Plasmids were constructed using standard molecular biological techniques [74]. The series of plasmid constructions and genetic manipulations used to create strains containing the components of the translocation [38] and direct-repeat recombination [75] assays were described previously. Construction of the *msh2*Δ [76], *msh3*Δ [76] and *rad1*Δ [77] alleles used in this study has been described previously.

The *msh6*Δ allele used in this study was constructed as follows: The *MSH6* coding sequence was amplified by PCR from wild-type yeast genomic DNA using the primers 5′ msh6 (5′-GCG TGA GCA GTA GCT GAT ACG CG-3′) and 3′ msh6 (5′-CCG GTG AGA AAC CCC ATT CTT GCC C-3′) to yield a 4,339 bp fragment spanning from 195 bp upstream of the start codon to 4,145 bp downstream. The *MSH6* fragment was digested with *Kpn* I and *Nru* I and inserted into pBluescript that had been cleaved in its polylinker sequence by digestion with *Kpn* I and *Eco* RV, generating pLAY403. A 3,129 bp segment of the *MSH6* coding sequence was removed from pLAY403 by digestion with *Nsi* I, the ends back-filled with T4 DNA polymerase, and ligated to a *Bgl* II linker to create pLAY406. pLAY406 was digested with *Bgl* II and the *Bam* HI/*Bgl* II *hisG-URA3-hisG* universal disruptor fragment inserted to create the plasmid pLAY411. The *msh6::hisG-URA3-hisG* disruption cassette was released from pLAY411 by digestion with *Pvu* II and used to transform yeast to uracil prototrophy. Disruption of the *MSH6* locus was confirmed by PCR and Southern blot analyses (G. Manthey and A. Bailis – unpublished observations).

The *msh2* missense alleles used in this study were derived from plasmids originally generated by Studamire et al. [61] to study the separation of the mismatch repair and non-homologous tail cleavage functions of Msh2. The plasmids pEAA72, pEAA76, pEAA79 and pEAA80 bearing *msh2-G855D*, *msh2-S561P*, *msh2-L574S* and *msh2-L584P* respectively were the generous gifts of Eric Alani. Each of the plasmids was digested with *Pvu* II to release 4.5 kb DNA fragments containing the *msh2* alleles. These fragments were cloned into the polylinker of the integrating plasmid, YIp356R [78], the kind gift of Phil Hieter, that had been digested with *Sma* I, generating the plasmids pLAY575 (*msh2-G855D*), pLAY582 (*msh2-L574S*), pLAY585 (*msh2-L584P*) and pLAY586 (*msh2-S561P*). pLAY575 and pLAY586 were linearized by digestion with *Bgl* II, and pLAY582 and pLAY585 were linearized with *Nhe* I prior to their use in transforming a recipient strain to uracil prototrophy. Insertion of the plasmids at the *MSH2* locus, creating duplications was confirmed by genomic Southern blot and PCR analyses (G. Manthey and A. Bailis – unpublished observations). Loss of the duplication to generate a single copy of the *MSH2* locus was selected on medium containing 5-fluoroorotic acid [79]. The presence of the missense mutations in Ura^−^ recombinants and in meiotic segregants was detected by a combination of PCR and restriction fragment length analysis. The presence of the *msh2-L584P* allele was detected by amplifying a 370 bp fragment with the primers msh2F-1502 (5′- GTA AAC TGG ATA CGT TGC G-3′) and msh2R-1872 (5′-GGC AAT CAC ATC TAA ATG CGC-3′) and digesting it with *Mnl* I, which generates 120 bp, 127 bp and 123 bp products with the mutant allele and 122 bp and 250 bp products with the wild-type allele. The presence of the *msh2-S561P* allele was detected by digesting the same PCR fragment with *Sal* I, which generates 179 bp and 191 bp fragments with the wild-type allele but cannot cut the mutant allele, leaving an intact 370 bp fragment. The presence of the *msh2-G855D* allele was detected with an allele-specific PCR protocol that used the primers 5′msh2-G855D (5′-GGT ATT TCA GAT CAG TCT TTT GA-3′) and 3′xbamsh2 (5′-CCC CCC TCT AGA GTA ATA TTA ATT ATA ACA ACA AGG-3′) and the following amplification conditions to produce a 360 bp product: An initial period of denaturation at 95°C for 10 min was followed by 38 cyles of denaturation at 94°C for 30 sec, annealing at 62°C for 30 sec, and elongation at 72°C for 30 sec, and was completed with a final period of elongation at 72°C for five min. An allele-specific PCR protocol was also used to detect the presence of the *msh2-L574S* allele which used the primers 5′msh2-L574S (5′-GTA TAT TTT TTA GTA CCA AAC AAT C-3′) and the 3′xbamsh2 primer described above with the following amplification conditions to produce a 1.2 kb product: An initial period of denaturation at 95°C for 10 min was followed by 38 cycles of denaturation at 94°C for 30 sec, annealing at 59°C for 30 sec, and elongation at 72°C for 1.5 min, and was completed with a final period of elongation at 72°C for five min.

### Spontaneous direct-repeat recombination (DRR) frequencies

Frequencies of DRR were determined as described previously [75]. Haploid strains containing a 415 bp duplication of a portion of the *HIS3* coding sequence flanking the plasmid YIp5 [80] that had been inserted into the *HIS3* locus [75] were streaked to single colonies on medium lacking uracil to maintain selection for the *URA3* marker on the plasmid. Single colonies were used to inoculate one ml synthetic complete medium lacking uracil (SC-Ura) cultures and grown to saturation at 30°C overnight. Appropriate dilutions were plated to YPD (1% yeast extract, 2% bacto peptone, 2% dextrose) agar to determine viability and complete synthetic medium lacking histidine (SC-His) to determine the number of recombination events restoring an intact *HIS3* gene in each culture. Recombination frequencies were determined by dividing the number of His^+^ recombinants by the number of viable cells plated. Median frequencies from at least 10 independent cultures were reported along with 95% confidence intervals determined from a table [81].

### HO-stimulated translocation frequencies

Frequencies of translocation formation in diploid strains subsequent to an HO endonuclease-mediated DSB adjacent to the *his3-*Δ*5′* substrate, or both the *his3-*Δ*5′* and *his3-*Δ*3′* substrates were determined as described previously [38]. The *his3-*Δ*5′* substrate lies at the *LEU2* locus on one copy of chromosome III, and the *his3-*Δ*3′* substrate lies at the *HIS3* locus on one copy of chromosome XV. Recombination to yield an intact *HIS3* gene is restricted to interactions between these substrates by the presence of a *his3-*Δ*200* allele [82] on the other copy of chromosome XV, from which the promoter and coding sequence of *HIS3* have been deleted. Briefly, one ml cultures of SC medium containing 3% glycerol and 3% lactate were inoculated with single colonies and incubated at 30°C overnight. Aliquots of cells were removed from each culture and plated on to YPD agar to determine viability prior to expression of HO endonuclease. An 100 µl aliquot of 20% galactose was added to each culture to induce expression of HO endonuclease from a *GAL1::HO* cassette integrated at the *TRP1* locus. After four hours of expression, appropriate dilutions of cells were plated to YPD agar to determine viability after HO cutting, and SC-His to select for recombinants. Translocation frequencies were determined by dividing the number of histidine prototrophic recombinant colonies by the number of viable cells plated. Median translocation frequencies from at least 10 independent cultures were reported and the 95% confidence intervals determined using a table [81]. Cells from selected His^+^ colonies were submitted to genomic Southern blot and chromosome blot analyses as described previously [38] and below.

### Plating efficiencies

Plating efficiencies were determined as described previously [38]. Cells containing HO cut sites at *his3-*Δ*3′*, *his3-*Δ*5′* and at the *MAT* loci on both copies of chromosome III that had been cultured in SC glycerol/lactate medium were collected before, and four hours after the addition of galactose. Cell number was assessed by hemocytometer count and appropriate dilutions plated onto YPD agar. The number of colonies was counted after two days of incubation at 30°C. Plating efficiency was calculated by dividing the number of colonies that arose on the plates by the number of cell bodies plated and multiplying the quotient by 100. The median plating efficiencies from at least seven independent trials were reported and the 95% confidence intervals determined from a table [81].

### Contour-clamped homogenous electric field (CHEF) analysis

Chromosomes from selected His^+^ recombinant colonies were prepared in agarose plugs using an established protocol [83]. Chromosomes were separated on 1% agarose gels with a Bio-Rad CHEF-DR II apparatus (BioRad, Hercules, CA) using parameters that were described previously [38]. The separated chromosomes were visualized after staining with 1 µg/ml ethidium bromide for 30 min and photographed. Ethidium stained chromosomes were irradiated with 60 mJoules of UV in a Stratagene Stratalinker 1800 to nick the DNA, and destained for 30 min in deionized, distilled water. The chromosomes were transferred from the gel to a nylon membrane (Hybond N^ = ^, GE Healthcare, Waukesha, WI) by electroblotting with a Genie Blotter apparatus (Idea Scientific Co., Minneapolis, MN). These were probed with a 1.8 kb *Bam* HI *HIS3* genomic clone corresponding to sequences from 469 bp upstream of the *HIS3* open reading frame to 634 bp downstream that had been labeled with ^32^P by random priming using a Megaprime DNA labeling kit (GE Healthcare). Blots were exposed to film, and the film developed using a Konica Minolta SRX-101A processor (Konica Minolta USA, Ramsey, NJ).

## Supporting Information

Table S1 T2 Frequencies - Translocation frequencies were determined as described in the Materials and Methods with substrates that possessed 60 bp or 300 bp of overlapping sequence. Median values from a minimum of 10 independent trials with each genotype are reported with 95% confidence intervals in parentheses. Fold differences from wild-type are reported in brackets. n. d. - not determined.
(0.06 MB DOC)

Table S2 Yeast strains used in this study - All strains were isogenic with W303-1A (*MATa ade2-1 can1-100 his3-11, 15 leu2-3, 112 trp1-1 ura3-1 rad5-G535R*)(73) except where noted and that all carried a wild type *RAD5* allele.
(0.07 MB DOC)
