# Supplementary material for: Msh2 Blocks an Alternative Mechanism for Non-Homologous Tail Removal during Single-Strand Annealing in Saccharomyces cerevisiae
Source: PLoS One. 2009 Oct 16;4(10):e7488. doi: 10.1371/journal.pone.0007488 (PMC2759526; doi:10.1371/journal.pone.0007488)
Supplement: Table S1 — T2 Frequencies - Translocation frequencies were determined as described in the Materials and Methods with substrates that possessed 60 bp or 300 bp of overlapping sequence. Median values from a minimum of 10 independent trials with each genotype are reported with 95% confidence intervals in parentheses. Fold differences from wild-type are reported in brackets. n. d. - not determined. (0.06 MB DOC) [file pone.0007488.s001.doc]

**Supplementary Table 1.** T2 Frequencies

| Genotype | Substrate Size | |
| --- | --- | --- |
|  | 60 bp | 300 bp |
| Wild-type | 9.3 x 10^-3^ (8.4, 14) [1] | 6.5 x 10^-2^ (5.5, 9.0) [1] |
| *rad1∆/rad1∆* | 1.0 x 10^-4^ (0.8, 1.6) [0.011] | 1.1 x 10^-4^ (0.7, 1.9) [0.0017] |
| *msh2∆/msh2∆* | 2.3 x 10^-4^ (1.3, 2.6) [0.025] | 5.3 x 10^-4^ (2.4, 10.6) [0.008] |
| *msh3∆/msh3∆* | n. d. | 1.1 x 10^-3^ (0.8, 3.0) [0.017] |
| *msh6∆/msh6∆* | n. d. | 9.3 x 10^-2^ (7.4, 12.1) [1.4] |
| *msh2-G855D/msh2-G855D* | 1.8 x 10^-3^ (1.4, 2.2) [0.19] | 4.5 x 10^-2^ (2.2, 4.9) [0.69] |
| *msh2-L574S/msh2-L574S* | 6.9 x 10^-4^ (5.0, 7.9) [0.074] | 5.3 x 10^-3^ (2.3, 6.3) [0.082] |
| *msh2-L584P/msh2-L584P* | 5.9 x 10^-4^ (4.2, 7.3) [0.061] | 3.5 x 10^-2^ (2.4, 4.5) [0.54] |
| *msh2-S561P/msh2-S561P* | 8.6 x 10^-3^ (6.3, 9.5) [0.93] | 4.5 x 10^-2^ (3.7, 6.3) [0.69] |
| *MSH2/msh2-G855D* | 3.1 x 10^-3^ (2.7, 3.9) [0.33] | n.d. |
| *MSH2/msh2-L574S* | 3.6 x 10^-3^ (2.7, 4.3) [0.39] | 2.7 x 10^-2^ (2.3, 2.9) [0.42] |
| *MSH2/msh2-L584P* | 2.4 x 10^-3^ (2.1, 4.4) [0.26] | 2.5 x 10^-2^ (2.4, 2.8) [0.39]. |
| *msh2-L574S/msh2-G855D* | 1.5 x 10^-3^ (1.2, 2.4) [0.16] | 3.2 x 10^-2^ (2.5, 4.4) [0.49] |
| *msh2-L574S/msh2-L584P* | 7.9 x 10^-4^ (3.3, 9.4) [0.085] | 2.2 x 10^-2^ (1.2, 2.9) [0.34] |
| *msh2∆/msh2∆ rad1∆/rad1∆* | 5.7 x 10^-4^ (3.3, 6.6) [0.061] | 4.3 x 10^-3^ (2.5, 5.3) [0.067] |
| *msh2-G855D/msh2-G855D*  *rad1∆/rad1∆* | 6.4 x 10^-4^ (5.1, 11.0) [0.069] | 5.5 x 10^-3^ (2.4, 7.9) [0.085] |
| *msh2-L574S/msh2-L574S*  *rad1∆/rad1∆* | 2.1 x 10^-4^ (1.4, 2.5) [0.023] | 4.1 x 10^-4^ (2.6, 8.5) [0.0063] |
| *msh2-L584P/msh2-L584P*  *rad1∆/rad1∆* | 4.5 x 10^-4^ (3.4, 5.7) [0.048] | 1.5 x 10^-3^ (1.2, 1.9) [0.023] |
