# Supplementary material for: Msh2 Blocks an Alternative Mechanism for Non-Homologous Tail Removal during Single-Strand Annealing in Saccharomyces cerevisiae
Source: PLoS One. 2009 Oct 16;4(10):e7488. doi: 10.1371/journal.pone.0007488 (PMC2759526; doi:10.1371/journal.pone.0007488)
Supplement: Table S2 — Yeast strains used in this study - All strains were isogenic with W303-1A (MATa ade2-1 can1-100 his3-11, 15 leu2-3, 112 trp1-1 ura3-1 rad5-G535R)(73) except where noted and that all carried a wild type RAD5 allele. (0.07 MB DOC) [file pone.0007488.s002.doc]

**Supplementary Table 2.** *Saccharomyces cerevisiae* strains used in this study.

| **Strain** | **Relevant Genotype** | **Reference** |
| --- | --- | --- |
| ABX1711 | *MAT****a****/α, his3-∆200/his3-∆3’-HOcs*  *leu2-3,112/ leu2::HOcs-his3∆5’_(300)_*  *trp1-1/trp1::GALHO::KANMX* | Pannunzio *et al.* 2008 |
| ABM150 | As ABX1711 except *rad1::LEU2/rad1::LEU2* | Pannunzio *et al.* 2008 |
| ABX1660 | As ABX1711 except *msh2::hisG::URA3::hisG/msh2::hisG::URA3::hisG* | Pannunzio *et al.* 2008 |
| ABX2856 | As ABX1711 except *msh3::hisG::URA3::hisG/msh3::hisG::URA3::hisG* | This study |
| ABX2657 | As ABX1711 except *msh6::hisG::URA3::hisG/msh6::hisG::URA3::hisG* | This study |
| ABX2305 | As ABX1711 except *msh2-G855D/msh2-G855D* | This study |
| ABX2404 | As ABX1711 except *msh2-L584P/msh2-L584P* | This study |
| ABX2406 | As ABX1711 except *msh2-L574S/msh2-L574S* | This study |
| ABX2407 | As ABX1711 except *msh2-S561P/msh2-L561P* | This study |
| ABX2639 | As ABX1711 except *msh2::hisG::URA3::hisG/msh2::hisG::URA3::hisG rad1::LEU2/rad1::LEU2* | This study |
| ABX2674 | As ABX1711 except *msh2-G855D/msh2-G855D rad1::LEU2/rad1::LEU2* | This study |
| ABX2680 | As ABX1711 except *msh2-L584P/msh2-L584P rad1::LEU2/rad1::LEU2* | This study |
| ABX2695 | As ABX1711 except *msh2-L574S/msh2-L574S rad1::LEU2/rad1::LEU2* | This study |
| ABX2727 | As ABX1711 except *msh2-L574S/msh2-G855D* | This study |
| ABX2728 | As ABX1711 except *msh2-L574S/msh2-L584P* | This study |
| ABM130 | *MAT****a****/α, his3-∆200/his3-∆3’-HOcs*  *leu2-3,112/ leu2::HOcs-his3∆5’_(60)_*  *trp1-1/trp1::GALHO::KANMX* | Pannunzio *et al.* 2008 |
| ABM131 | As ABM130 except *rad1::LEU2/rad1::LEU2* | Pannunzio *et al.* 2008 |
| ABM158 | As ABM130 except *msh2::hisG::URA3::hisG/msh2::hisG::URA3::hisG* | Pannunzio *et al.* 2008 |
| ABX2319 | As ABM130 except *msh2-G855D/msh2-G855D* | This study |
| ABX2412 | As ABM130 except *msh2-L574S/msh2-L574S* | This study |
| ABX2425 | As ABM130 except *msh2-L584P/msh2-L584P* | This study |
| ABX2473 | As ABM130 except *msh2-S561P/msh2-S561P* | This study |
| ABX2672 | As ABM130 except *msh2::hisG::URA3::hisG/msh2::hisG::URA3::hisG rad1::LEU2/rad1::LEU2* | This study |
| ABX2673 | As ABM130 except *msh2-G855D/msh2-G855D rad1::LEU2/rad1::LEU2* | This study |
| ABX2694 | As ABM130 except *msh2-L574S/msh2-L574S rad1::LEU2/rad1::LEU2* | This study |
| ABX2716 | As ABM130 except *MSH2/msh2-S561P* | This study |
| ABX2718 | As ABM130 except *MSH2/msh2-L574S* | This study |
| ABX2720 | As ABM130 except *MSH2/msh2-L584P* | This study |
| ABX2729 | As ABM130 except *msh2-L574S/msh2-G855D* | This study |
| ABX2730 | As ABM130 except *msh2-L574S/msh2-L584P* | This study |
| ABX2734 | As ABM130 except *MSH2/msh2-G855D* | This study |
| ABX2749 | As ABX1711 except *MSH2/msh2-L584P* | This study |
| ABX2751 | As ABX1711 except *MSH2/msh2-L574S* | This study |
| ABX2756 | As ABM130 except *msh2-L584P/msh2-L584P rad1::LEU2/rad1::LEU2* | This study |
| ABX1235-16D | *MAT****a*** *his3-URA3-his3_(415)_* | This study |
| ABX2699-17X | As ABX1235-1D except *msh2::hisG::URA3::hisG* | This study |
| ABX2520 | *MAT****a****/α, his3-∆200/his3-∆3’-HOcs_(MUT)_*  *leu2-3,112/leu2::HOcs-his3∆5’_(60)_*  *trp1-1/trp1::GALHO::KANMX* | This study |
| ABX2521 | *MAT****a****/α, his3-∆200/his3-∆3’-HOcs_(MUT)_*  *leu2-3,112/leu2::HOcs-his3∆5’_(300)_*  *trp1-1/trp1::GALHO::KANMX* | This study |
| ABX2693 | As ABX2521 except *msh2::hisG::URA3::hisG/msh2::hisG::URA3::hisG* | This study |
| ABX2528 | As ABX2520 except *msh2::hisG::URA3::hisG/msh2::hisG::URA3::hisG* | This study |
